# Untargeted Metabolomics Analysis of the Serum Metabolic Signature of Childhood Obesity

**DOI:** 10.3390/nu14010214

**Published:** 2022-01-04

**Authors:** Lukasz Szczerbinski, Gladys Wojciechowska, Adam Olichwier, Mark A. Taylor, Urszula Puchta, Paulina Konopka, Adam Paszko, Anna Citko, Joanna Goscik, Oliver Fiehn, Sili Fan, Anna Wasilewska, Katarzyna Taranta-Janusz, Adam Kretowski

**Affiliations:** 1 Department of Endocrinology, Diabetology and Internal Medicine, Medical University of Bialystok, Sklodowskiej-Curie 24A, 15-276 Bialystok, Poland; puchta.urszula@gmail.com (U.P.); adam.kretowski@umb.edu.pl (A.K.); 2 Clinical Research Centre, Medical University of Bialystok, Sklodowskiej-Curie 24A, 15-276 Bialystok, Poland; gladys.wojciechowska@umb.edu.pl (G.W.); adam.olichwier@umb.edu.pl (A.O.); mark.taylor@ucsf.edu (M.A.T.); paulina.konopka@umb.edu.pl (P.K.); adam.paszko@umb.edu.pl (A.P.); anna.citko@umb.edu.pl (A.C.); joanna.goscik@umb.edu.pl (J.G.); 3 Helen Diller Family Comprehensive Cancer Center, University of California at San Francisco, 505 Parnassus Ave., San Francisco, CA 94143, USA; 4 West Coast Metabolomics Center, UC Davis Genome Center, University of California, Davis, 451 Health Sciences Drive, Davis, CA 95616, USA; ofiehn@ucdavis.edu (O.F.); slfan@ucdavis.edu (S.F.); 5 Department of Pediatrics and Nephrology, Medical University of Bialystok, 15-274 Bialystok, Poland; annwasil@interia.pl (A.W.); katarzyna.taranta@wp.pl (K.T.-J.)

**Keywords:** childhood obesity, untargeted metabolomics, lipidomics, obesity pathogenesis, obesity biomarkers, obesity pathomechanisms

## Abstract

Obesity rates among children are growing rapidly worldwide, placing massive pressure on healthcare systems. Untargeted metabolomics can expand our understanding of the pathogenesis of obesity and elucidate mechanisms related to its symptoms. However, the metabolic signatures of obesity in children have not been thoroughly investigated. Herein, we explored metabolites associated with obesity development in childhood. Untargeted metabolomic profiling was performed on fasting serum samples from 27 obese Caucasian children and adolescents and 15 sex- and age-matched normal-weight children. Three metabolomic assays were combined and yielded 726 unique identified metabolites: gas chromatography–mass spectrometry (GC–MS), hydrophilic interaction liquid chromatography coupled to mass spectrometry (HILIC LC–MS/MS), and lipidomics. Univariate and multivariate analyses showed clear discrimination between the untargeted metabolomes of obese and normal-weight children, with 162 significantly differentially expressed metabolites between groups. Children with obesity had higher concentrations of branch-chained amino acids and various lipid metabolites, including phosphatidylcholines, cholesteryl esters, triglycerides. Thus, an early manifestation of obesity pathogenesis and its metabolic consequences in the serum metabolome are correlated with altered lipid metabolism. Obesity metabolite patterns in the adult population were very similar to the metabolic signature of childhood obesity. Identified metabolites could be potential biomarkers and used to study obesity pathomechanisms.

## 1. Introduction

The prevalence of pediatric obesity is rising globally [1,2]. Worldwide, over 379 million children and adolescents are overweight or obese [1]. Obesity during developmental years tends to persist into adulthood and increases the risk for other health disorders [3,4]. For example, children with obesity have a higher risk of early puberty, cardiovascular and pulmonary diseases at a younger age, and psychosocial issues [5,6]. The obesity epidemic among children is leading to an increased prevalence of impaired fasting glucose (IFG), impaired glucose tolerance (IGT), and even full-blown type 2 diabetes (T2D) in early adulthood [7,8]. Together, these conditions profoundly lower the quality of life of children, affecting future health as adults. A better understanding of the mechanisms underpinning the development of obesity and its early consequences, preferably before symptoms are evident, is crucial for developing new therapies.

Metabolomics is a technology for profiling and measuring the levels of low-molecular-weight metabolites (<1500 Da) in various systems, from cells to whole organisms [9,10]. There are two major approaches for metabolomics: untargeted and targeted metabolite profiling. The untargeted approach involves agnostic profiling of all metabolites within a sample, which aims to identify diverse metabolites to generate hypotheses. Targeted profiling focuses on the quantitative measurement of specific metabolites and testing hypotheses. Both approaches can be used to identify biomarkers to unveil the molecular mechanisms of complex diseases, for monitoring diseases, and risk evaluation [11,12].

Metabolomics has been used to study the metabolic signature of obesity, characterize the differential responses to dietary or weight loss interventions, predict intervention outcomes, and study the effects of specific dietary patterns on obesity-related metabolites [12]. Metabolomics studies have also been carried out on specific cohorts, including pediatric obesity [13,14,15,16]. Children with elevated concentrations of stearate, oleate, or palmitate at birth, determined from umbilical cord samples, were found to develop obesity by 3−5 years old [16]. Differential concentrations of plasma metabolites were also seen in Hispanic children with obesity [15]. For example, children with obesity had increased plasma concentrations of leucine, isoleucine, and valine but lower concentrations of asparagine, aspartate, glycine, and serine [15]. Targeted metabolomics has also been performed, such as targeted profiling of serum acyl-alkyl phosphatidylcholines and urine steroid profiles in children with obesity [13,14]. However, studies on metabolomics in pediatric populations remain limited. To better understand the pathogenesis of obesity, we must validate previous findings and evaluate metabolic alterations in obese children yet to develop disease symptoms.

In this study, we used three untargeted metabolomics platforms: gas chromatography–mass spectrometry (GC–MS), hydrophilic interaction liquid chromatography coupled to mass spectrometry (HILIC LC–MS/MS), and lipidomics, to investigate differences in the serum metabolome between children with and without obesity. We aimed to understand the metabolic signature of pediatric obesity before symptoms are evident, specifically for children in Eastern European populations. We also attempted to identify potential obesity-associated metabolite biomarkers that differentiate children with and without obesity. The findings will help us better understand the development of obesity and could assist in identifying new molecular targets for the treatment of the disease.

## 2. Materials and Methods

### 2.1. Studied Cohort

Untargeted metabolomic profiling was performed on fasting serum samples from 27 obese Caucasian children and adolescents and 15 sex- and age-matched normal-weight children. They were recruited from the patients referred to the Department of Pediatrics and Nephrology, Medical University of Bialystok Children’s Clinical Hospital (Bialystok, Poland), between 2017 and 2019. Patients who met all the following inclusion criteria were enrolled into the obesity group: (1) age 5−18 years, (2) obesity defined as body mass index (BMI) > 97th percentile of Polish population-specific data [17], (3) normal clinical examination, (4) no clinical or laboratory signs of infection, (5) normal levels of cortisol and thyroid-stimulating hormone, (6) normal electrocardiogram, and (7) signed informed consent. The control group consisted of healthy, age- and sex-matched children admitted to the Department due to suspicion of kidney or urinary tract anomalies. Children in the control group were qualified based on medical history and screening tests. The exclusion criteria in the obesity and the control groups were any chronic disease (except obesity in the obesity group) or pharmacotherapy. The Bioethics Committee of the Medical University of Bialystok approved this study. Written informed consent was obtained from all subjects and their parents.

### 2.2. Clinical Features 

Height was measured to the nearest centimeter using a rigid stadiometer. Undressed weight, in the fasted state, was measured to the nearest 0.1 kg using a calibrated balance scale. BMI was calculated as body mass (kg) divided by height (m) squared. The BMI z-score was calculated using Cole’s international childhood BMI cut-offs [18,19]. Blood pressure was measured using an automated oscillometric device validated for use in children. Four cuff sizes were available (child’s cuff, small adult cuff, adult cuff, and large adult cuff). The appropriate cuff size (bladder width at least 40% of arm circumference and length 80−100% of arm circumference) was determined by measuring the mid-upper arm circumference. Systolic blood pressure (SBP) and diastolic blood pressure (DBP) were measured in the non-dominant arm in triplicate, at 3 min intervals after a 5−10 min rest in the sitting position with the arm and back supported. The average values of the second and third measurements were used for subsequent analyses.

Venous blood samples were collected at 7:00 a.m., after overnight fasting for at least 10 h. Following coagulation at room temperature, blood samples were centrifuged for 10 min at 8000 rpm. Aliquoted serum samples were stored at −80 °C and thawed at room temperature for the metabolomics assay. Collected blood samples were also used to measure total serum triglycerides (TG), total cholesterol (TChol), high-density lipoprotein cholesterol (HDL) and low-density lipoprotein cholesterol (LDL), urea, uric acid, creatinine, and plasma glucose concentrations, using colorimetric Cobas c111 kits according to manufacturer’s protocols (Roche Diagnostics, Basel, Switzerland). Serum thyroid-stimulating hormone (TSH), cortisol, and insulin were measured by electrochemiluminescence immunoassays on a Cobas e411 analyzer (Roche Diagnostics).

### 2.3. Metabolomics Data Acquisition and Processing

Untargeted metabolomic profiling was carried out in the West Coast Metabolomics Center at the University of California Davis, USA. Analysis was performed as previously described [20,21,22,23] along with detailed descriptions of internal standards [23]. Briefly, serum samples were extracted for primary metabolites, complex lipids, and biogenic amines. For primary metabolites, 20 μL serum samples were extracted with 1 mL degassed cold (−20 °C) acetonitrile (ACN):isopropanol:water (3:3:2; *v*/*v*/*v*) solution. The supernatant (500 μL) mixed with 500 μL of ACN:water (1:1) was dried and derivatized using methoxyamine hydrochloride and N-methyl-N-trimethylsilyl trifluoroacetamide (MSTFA). A mixture of fatty acid methyl esters (FAMEs) from C8 to C30 was added as internal standards. For lipids and biogenic amines, 40 μL serum samples were extracted with a biphasic solvent system of 300 μL of cold methanol containing internal lipid standards, 1000 μL methyl tert-butyl ether (MTBE) containing CE (22:1) internal standard, and 250 μL of water. For the LC–MS analysis, the organic phase (100 μL) was vacuum-dried and reconstituted in 100 μL of methanol:toluene (9:1, *v*/*v*) containing 1-cyclohexyl-ureido-3-dodecanoic acid (CUDA; 150 ng/mL) internal standard. For HILIC analysis, the aqueous phase was vacuum-dried and rinsed with ACN:water (1:1, *v*/*v*) for protein removal, then vacuum-dried again. Sample reconstitution was performed using ACN:water (80:20, *v*/*v*) solution containing CUDA (150 ng/mL) and deuterated internal standards. 

GC–MS was performed on 0.5 μL primary metabolite extracts on an Agilent 6890 GC-LECO Pegasus III TOF instrument (Agilent, Santa Clara, CA, USA) equipped with a Cooled Injection System (CIS4), an Automated Linear Exchange system (ALEX), and a Multi Purpose Sampler (MPS, all Gerstel) [20,23]. Briefly, the injector conditions were as follows: initial and final temperatures of 50 °C and 275 °C, respectively, at a rate of 12 °C/s, volumes of 0.5 µL, and splitless mode with a purge time of 25 s. An Rtx-5Sil MS column (30 m length, 0.25 mm i.d., 0.25 μM 95% dimethyl 5% diphenyl polysiloxane film) with a 10 m integrated guard column. The GC temperature started at 50 °C for 1 min, then increased to 330 °C at 20 °C/min, and was held for 5 min. The transfer line and ion source temperatures were 280 °C and 250 °C, respectively. The acquisition rate was 17 spectra/s at a detector voltage of 1525 V and a scan mass range of 85–500 Da. ChromaTOF software (v2.32) was used for peak deconvolution and picking, while BinBase [24] was used to identify metabolites [20,23]. 

Complex lipid separation was carried out using a Waters Acquity UPLC CSH C18 column (100 × 2.1 mm; 1.7 μm; Waters) with an additional Acquity UPLC CSH C18 VanGuard precolumn (5 × 2.1 mm; 1.7 μm), maintained at 65 °C. The mobile phase for positive mode was (A) ACN:water (60:40, *v*/*v*) with ammonium formate (10 mM) and formic acid (0.1%) and (B) 2-propanol:ACN (90:10, *v*/*v*) with ammonium formate (10 mM) and formic acid (0.1%). For negative mode, the mobile phase was (A) ACN:water (60:40, *v*/*v*) with ammonium formate (10 mM) and (B) 2-propanol:ACN (90:10, *v*/*v*) with ammonium formate (10 mM). A 2 µL sample was injected in each case. The separation gradient was 0 min 15% B, 0−2 min 30% B, 2−2.5 min 48% B, 2.5−11 min 82% B, 11−11.5 min 99% B, 11.5−12 min 99% B, 12−12.1 min 15% B, and 12.1−15 min 15% B. An Agilent 6550 QTOF (Agilent, Santa Clara, CA, USA) with a jet stream electrospray source was used with the following parameters: mass range m/z 50−1700, capillary voltage ± 3 kV, nozzle voltage ± 1 kV, gas temperature 200 °C, drying gas (nitrogen) flow rate 14 L/min, nebulizer gas (nitrogen) 35 psi, sheath gas temperature 350 °C, sheath gas flow rate (nitrogen) 11 L/min, acquisition rate two spectra/s. Lipid identification was performed by collecting MS/MS spectra at a collision energy of 20 eV. The acquisition rates for MS1 and MS/MS were 10 spectra/s (100 ms) and 13 spectra/s (77 ms), respectively.

HILIC-Q Exactive MS/MS data acquisition was performed for biogenic amines. Samples (1 μL) were injected onto a Waters Acquity UPLC BEH Amide column (150 × 2.1 mm; 1.7 μm; Waters) coupled to an Acquity UPLC BEH Amide VanGuard precolumn (5 × 2.1 mm; 1.7 μm), maintained at 45 °C with a flow rate of 0.4 mL/min. The mobile phases were (A) water with ammonium formate (10 mM) and formic acid (0.125%) and (B) ACN:water (95:5, *v*/*v*) with ammonium formate (10 mM) and formic acid (0.125%). Gradient elution was performed from 100% (B) at 0−2 min to 70% (B) at 7.7 min, 40% (B) at 9.5 min, 30% (B) at 10.25 min, 100% (B) at 12.75 min, isocratic until 16.75 min. Positive and negative mode electrospray ionization was performed using a Q Exactive Orbitrap MS, with the following parameters: mass range 60−900 m/z, sheath gas flow rate 60, aux gas flow rate 25, capillary temperature 300 °C, aux gas heater temperature 370 °C. MSMS parameters were MS1 accumulation time 100 ms at 60,000 resolution, MS2 accumulation time 50 ms, isolation window 1.0 m/z, and normalized collision energies 20, 30, and 40. 

Lipid and biogenic amine data processing was performed using MS-DIAL [25] for deconvolution, peak picking, alignment, and identification. Metabolite annotation was performed following Metabolomics Standard Initiative (MSI) guidelines [26,27], using in-house mzRT libraries and MS/MS spectral databases [28,29,30,31].

### 2.4. Metabolomics Data Normalization and Analysis

A total of 726 unique metabolites, obtained from the three profiling methods, were identified across 41 samples. Data normalization and analysis were carried out using MetaboAnalyst 5.0 [32]. Data exclusion was performed for metabolites with constant values across metabolites and interquartile filtering. The process resulted in 538 metabolites for downstream analysis. Missing values were mean imputed, and normalization was performed using log10 transformation. 

For univariate analysis, fold changes and T-test values were calculated, then multiple testing correction was performed based on false discovery rate (FDR). ROC-curve analysis was also carried out for each metabolite, and 95% confidence intervals were calculated using bootstrapping with 500 permutations. Multivariate exploratory analysis was performed using principal component analysis (PCA) and orthogonal projections to latent structures discriminant analysis (OPLS-DA), as implemented in MetaboAnalyst. Permutation testing for OPLS-DA was applied to evaluate model stability to parameter addition. Linear support vector machine (SVM) classifiers were built to predict group class using Monte-Carlo cross-validation (MCCV) and balanced subsampling. Six SVMs with an increasing number of metabolites (maximum 100) were compared. Model evaluation was performed using ROC curves, and biomarker identification was achieved using the feature ranking method implemented in SVM. 

## 3. Results

### 3.1. Clinical Characteristics of the Studied Population

The demographic and clinical characteristics of study participants are presented in Table 1. The groups were matched for age and sex. As anticipated, subjects with obesity had significantly higher body mass index (both BMI and BMI Z-score) after multiple testing corrections. No significant differences were observed in fasting plasma glucose, lipids (total cholesterol, HDL, LDL), creatinine, uric acid, TSH, and blood pressure values between studied groups (*p* > 0.05).

### 3.2. Metabolomics Differences between Studied Groups

#### 3.2.1. Univariate Analysis

**Figure 1:**
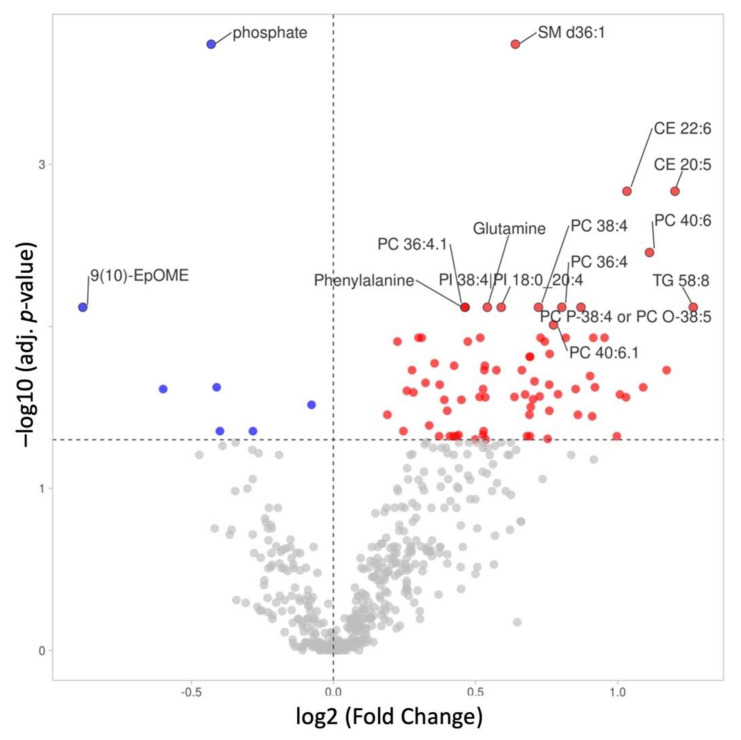
Volcano plot of metabolites across groups, with log-transformed adjusted *p*-values and fold changes. Red circles represent metabolites with increased expression in the obesity group. Blue circles represent metabolites with decreased expression in subjects with obesity. Grey circles represent non-significant metabolites (adjusted *p*-values ≥ 0.05). The top 15 top significant metabolites are labeled. SM, sphingomyelin; PC, phosphatidylcholine; CE, cholesteryl ester; TG, triacylglycerol; 9,10-EpOME, 9,10-epoxyoctadecenoic acid; PI, phosphatidylinositol.

We identified 726 metabolites from three untargeted metabolomics platforms (GC–MS, HILIC, and lipidomics). A total of 162 metabolites were significantly differentially expressed between groups (83 after correction for multiple testing; Figure 1, Table S1). Among significantly different metabolites after *p*-value adjustment, 76 metabolites showed significantly higher concentrations in children with obesity, with fold changes up to 2.4. By contrast, seven metabolites had lower concentrations, with fold changes reaching 0.54 (Figure 1, Table S1).

**Figure 2:**
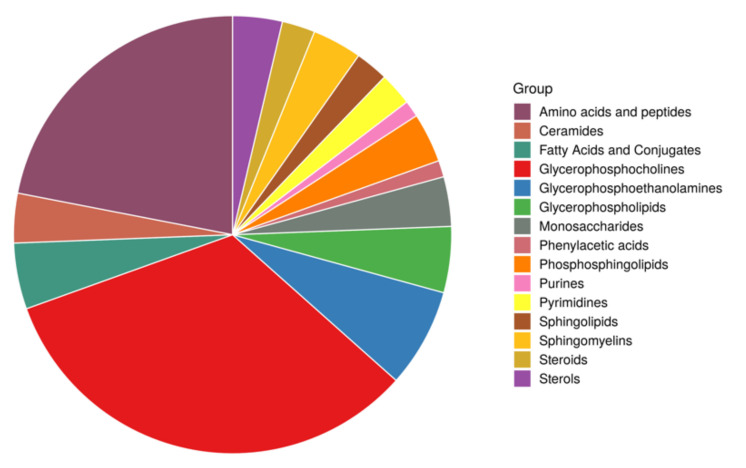
Metabolite classes with significantly different concentrations between patient groups.

Among metabolites that were significantly differentially abundant in studied groups, lipids and amino acids were among the top chemical classes (Figure 2). Children with obesity had higher concentrations of lipid metabolites, including phosphatidylcholines (PCs), cholesteryl esters (CEs), sphingomyelins (SMs), phosphatidylinositols (PIs), and triglycerides (TGs). We also found significantly higher concentrations of amino acids, including branched-chain amino acids (BCAA) leucine and valine.

**Figure 3:**
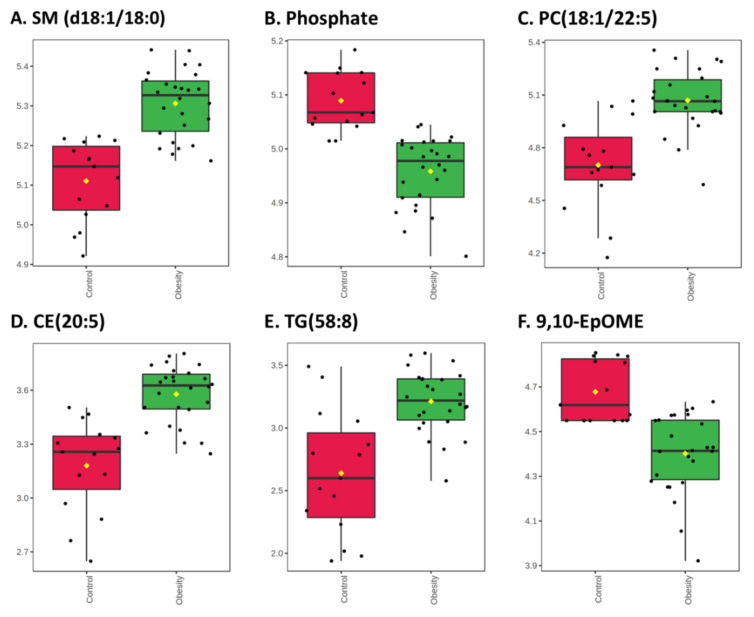
Box-plots of selected metabolites with significantly different concentrations between children with (green) and without (red) obesity. Fold changes and *p*-values are provided in Table S1. (**A**) Sphingomyelin (SM) d36:1 (d18:1/18:0); (**B**) Phosphate; (**C**) Phosphatidylcholine (PC) 40:6 (18:1/22:5); (**D**) Cholesteryl eicosapentaenoic acid (CE (20:5); (**E**) Triacylglycerol (TG) 58:8; (**F**) 9,10-epoxyoctadecenoic acid (9,10-EpOME).

**Figure 4:**
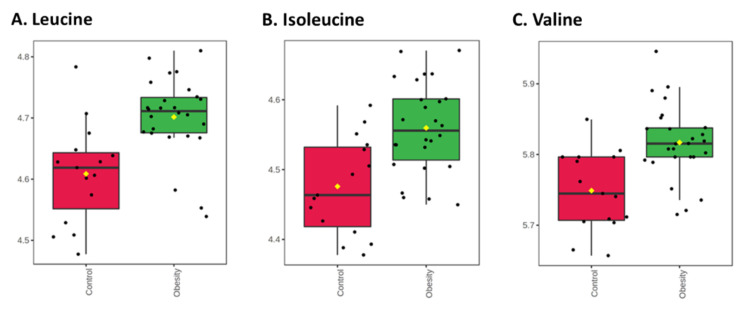
Box-plots of BCAAs with significantly different concentrations between children with (green) and without (red) obesity. Fold changes and *p*-values are provided in Table S1. (**A**) Leucine; (**B**) Isoleucine; (**C**) Valine.

The distributions of selected metabolites from each class are shown in Figure 3 and Figure 4. The top significant metabolites were SM d36:1 (d18:1/18:0; FC = 1.56, adj. *p*-value = 0.00018) and phosphate (FC = 0.74, adj. *p*-value = 0.00018), with a higher and lower concentration in children with obesity, respectively (Figure 3A,B). 

Most PCs had significantly higher concentrations in children with obesity, and the largest fold change was seen for PC 40:6 (18:1/22:5; FC = 2.16, adj. *p* = 0.0035; Figure 3C). However, three PCs had lower concentrations in those with obesity, with the largest reduction observed for PC (o-18:0/18:2; FC = 0.66, adj. *p* = 0.024, Table S1). 

Children with obesity had higher concentrations of CE, with the largest change seen in CE (20:5; FC = 2.3; adj. *p* = 0.0015; Figure 3D).

Among TGs, the largest increase was seen in TG (58:8; FC = 2.4, adj. *p* = 0.0076; Figure 3E). All significant TGs had higher concentrations in children with obesity. 

Interestingly, the metabolite with the biggest reduction in concentration in obese subjects, compared to the non-obese group, was 9,10-epoxyoctadecenoic acid (9,10-EpOME; FC = 0.54, adj. *p* = 0.0076, Figure 3F). 

BCAA concentrations were significantly higher in children with obesity (Figure 4), as seen for leucine (FC = 1.23; adj. *p* = 0.012), isoleucine (FC = 1.21; adj. *p* = 0.019), and valine (FC = 1.17; ad. *p* = 0.012).

Fold changes and *p*-values for all detected metabolites are listed in Table S1.

**Figure 5:**
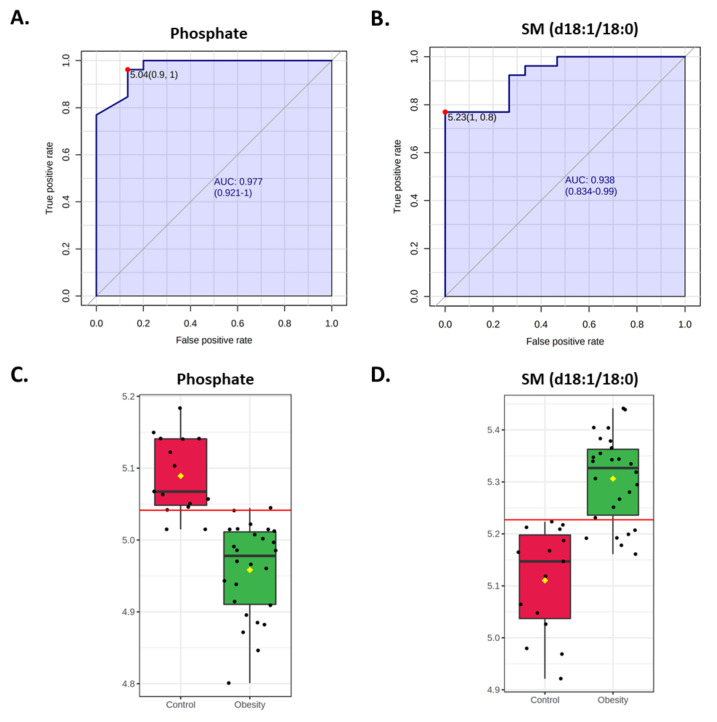
Univariate ROC curve results for phosphate and sphingomyelin SM (d18:1/18:0). (**A**,**B**) ROC curves for (**A**) phosphate (**A**) and (**B**) SM (d18:1/18:0). Sensitivity and specificity are shown on the y- and x-axes, respectively. The area-under-the-curve (AUC) is in blue, and 95% CIs are shown. (**C**,**D**) Box-plots of (**C**) phosphate and (**D**) SM (d18:1/18:0) between children with (green) and without (red) obesity. The red horizontal line represents the optimal cut-off.

Univariate ROC curve analysis was performed for each metabolite. A total of 541 and 37 metabolites have an area under the ROC curve (AUC) of at least 0.50 and 0.80, respectively (Table S2). The top two metabolites that can discriminate the groups with the highest accuracy are phosphate (AUC = 0.977, 95% CI = 0.921–1) and SM (d18:1/18:0; AUC = 0.938, 95%CI = 0.834–0.99; Figure 5; Table S2). These metabolites are also the most significant. 

#### 3.2.2. Multivariate Analysis

**Figure 6:**
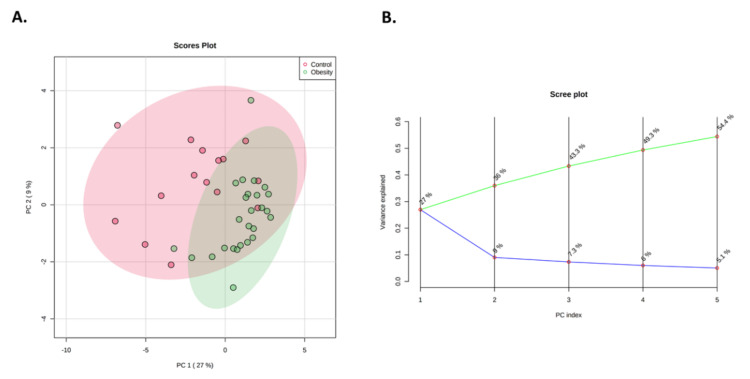
PCA analysis between patient groups. (**A**) Two-dimensional (2D) score plots between PC1 and PC2. Patients with obesity are shown in green, and those without obesity are shown in red. (**B**) Scree plot showing the variance explained by PCs 1−5.

Two multivariate exploratory analyses were performed to evaluate separation between patient groups; unsupervised PCA and supervised OPLS-DA. PCA showed that serum metabolites clustered children with obesity together, but there were some overlaps with children without obesity (Figure 6). The first principal component (PC1) explains 27% of the overall variability and adding five PCs explains 54.4% (Figure 6). OPLS-DA was validated with permutations test (*n* = 2000). 

**Figure 7:**
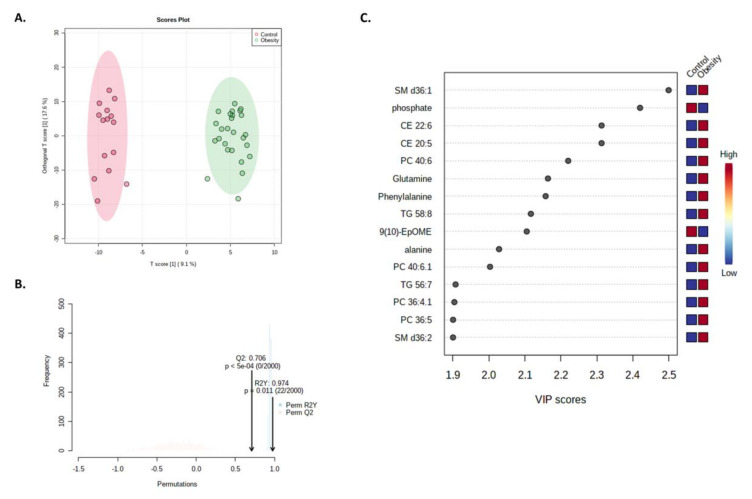
OPLS-DA analysis between patient groups. (**A**) Score plot of all metabolite features. (**B**) Permutation analysis with observed and cross-validated R2Y and Q2 coefficients. (**C**) Important metabolites identified by OPLS-DA. Colored boxes on the right indicate metabolite concentrations in each patient group. SM, sphingomyelin; PC, phosphatidylcholine; CE, cholesteryl ester; TG, triacylglycerol; 9,10-EpOME, 9,10-epoxyoctadecenoic acid.

A supervised OPLS-DA model was created to identify significantly differentially abundant metabolites between groups and validated using permutation tests (Figure 7). A random permutations test (*n* = 2000) resulted in an interpretation rate (R2) and prediction ability (Q2) of 0.974 and 0.706, respectively (Figure 7). According to the variable importance in projection (VIP) scores, the top 10 metabolites were SM d36:1 (SM (d18:1/18:0)), phosphate, CE 22:6, CE 20:5, PC 40:6 (PC(18:1/22:5)), L-glutamine, L-phenylalanine, TG 58:8, 9,10-EpOME (9,10-epoxyoctadecenoic acid) and L-alanine (Figure 6B). These metabolites displayed significantly different concentrations between groups, with fold changes >1.2 or <0.8 (Table S1).

**Figure 8:**
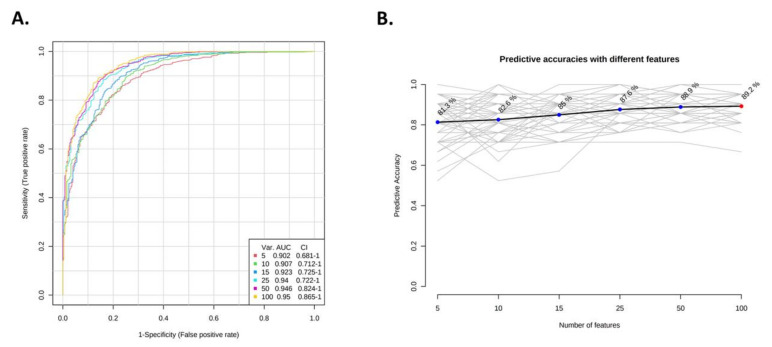
Model performance of six SVM classifiers with an increasing number of metabolites. (**A**) ROC curves for each SVM classifier, based on average cross-validation performance. AUCs and 95% CIs are presented in the figure legend. (**B**) Predictive accuracy for each SVM. The model with the highest accuracy is highlighted in red.

Support vector machine (SVM) classifiers were built to evaluate the ability of metabolites to predict patient groups (Figure 8). SVM with only five metabolites had a good AUROC (0.902, 95% CI = 0.681-1), and increasing the number of metabolites to 25 improved the AUROC to 0.94 (95% CI = 0.722-1). Further addition of metabolites only slightly improved AUROC (AUROC with 100 metabolites = 0.95, 95% CI = 0.865-1; Figure 8A). Similar observations were made for prediction accuracies (Figure 8B), where adding 20 metabolites increased accuracy by 6%, compared to the model with five metabolites. However, using 100 metabolites only improved accuracy by 1.6%, compared to SVM with 25 metabolites. 

**Figure 9:**
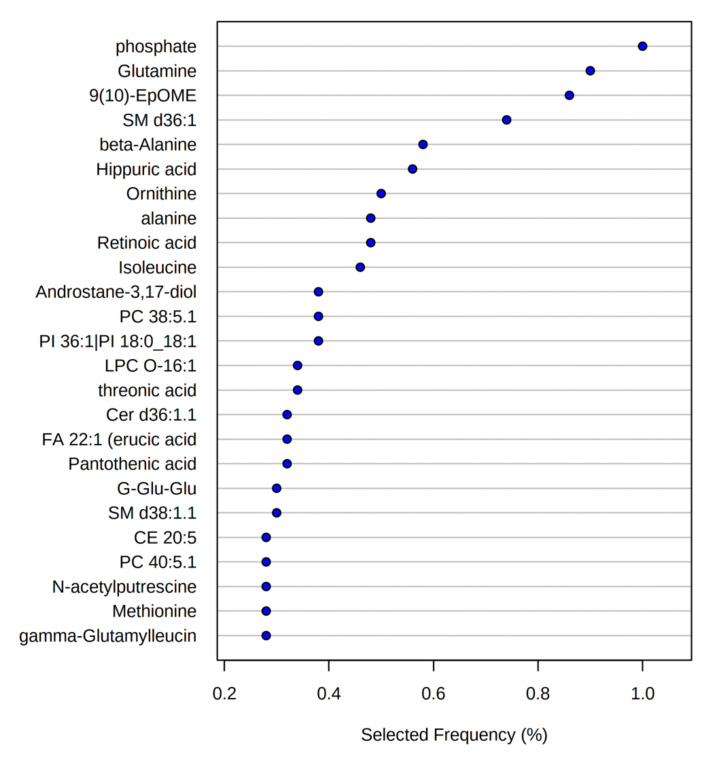
Variable importance from the SVM model with 25 metabolites. Metabolites are ranked from most to least important. The colored boxes on the right indicate metabolite concentrations in each patient group. SM, sphingomyelin; PC, phosphatidylcholine; PI, phosphatidylinositol; CE, cholesteryl ester; TG, triacylglycerol; 9,10-EpOME, 9,10-epoxyoctadecenoic acid; LPC, lysophosphatidylcholine; FA, fatty acid; Cer, ceramide; G-Glu-Glu, gamma-glutamyl-glutamic acid.

Variables selected in the SVM model with 25 metabolites are shown in Figure 9. Most of these metabolites had significantly different concentrations between patient groups (unadj. *p*-values ≤ 0.05, Table S1). Interestingly, two non-significant metabolites were selected in the model: PI 36:1(18:0/18:1; FC = 0.844, *p*-value = 0.178) and SM d38:1.1 (FC = 1.077, *p*-value = 0.086).

### 3.3. Clinical Correlations of Selected Metabolites

**Figure 10:**
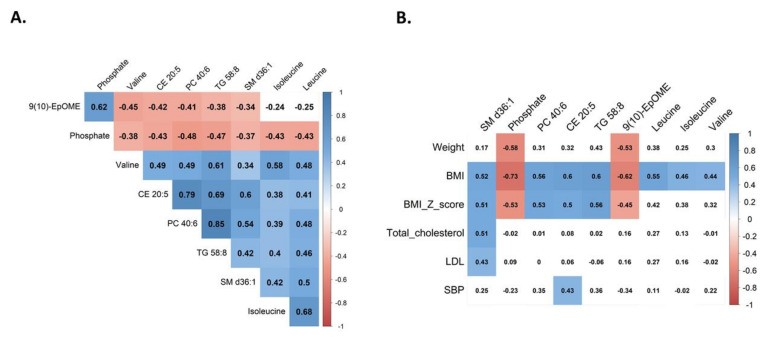
Heatmap of the Pearson correlation coefficient matrix: (**A**) Correlations between selected metabolites and (**B**) between selected metabolites and clinical variables. Red and blue indicate negative and positive correlations, respectively. Color intensity indicates the absolute correlation value. Empty cells indicate non-significant correlations (adjusted *p*-values > 0.05). SM, sphingomyelin; PC, phosphatidylcholine; CE, cholesteryl ester; TG, triacylglycerol; 9,10-EpOME, 9,10-epoxyoctadecenoic acid; BMI, body mass index; LDL, low-density lipoprotein cholesterol; SBP, systolic blood pressure.

The top metabolites from univariate analyses had moderate to high correlations with BMI and several other clinical variables (Figure 10). Except for phosphate and 9,10-EpOME, all these metabolites were significantly positively correlated with BMI. This relationship is also shown in Figure 3. Similar correlations were seen for BMI Z-scores, but the positive correlations between Z-scores and leucine, isoleucine, and valine were not significant after FDR correction. Weight showed a significantly negative correlation with phosphate and 9,10-EpOME. SM d36:1 was moderately correlated with total cholesterol and LDL. CE 20:5 was moderately correlated with systolic blood pressure. The metabolites had low to moderate relationships with other clinical variables, such as age, glucose, TGs, and HDL, but the correlations were not significant after FDR correction (Figure S1). 

Moreover, the top metabolites had moderate to high correlations with each other (Figure 10). Phosphate and 9,10-EpOME were negatively correlated with all metabolites but not between each other. The rest were mainly positively correlated. These relationships were significant after FDR correction, except for correlations between isoleucine and SM d36:1 and leucine (Figure 10A). 

## 4. Discussion

Obesity is a serious and growing health problem worldwide that affects adults and children. However, the exact mechanism involved in obesity development in children is not well known. Thus, in our study, we attempted to determine the metabolomic signature of obesity in children yet to develop metabolic consequences of the disease. Moreover, we evaluated whether metabolite patterns of obesity in pediatric populations are similar to those observed in adults.

Obesity alters lipid levels through processes linked to lipid metabolism, such as lipolysis, oxidation of FAs, and lipogenesis [33], which may lead to the onset and development of several metabolic disorders [34]. Additionally, many lipids function as signaling molecules involved in inflammation pathways or insulin resistance [35,36], thereby mediating the development of obesity complications like T2D or cardiovascular disease [37]. We showed that lipids are the most crucial group of altered metabolites in obese children compared to the non-obese control group. This observation aligns with the other metabolomics studies on obesity in adults [38,39] and limited data on pediatric populations [40,41]. Obesity is associated with elevated levels of TGs [42,43], sphingolipids [44], SM [45], CE [43], and phospholipids (PLs) [46] in adult obese patients. Similarly, elevated TG levels were observed in children from Taiwan [47] and Denmark [48]. Elevated TG levels in plasma is one of the most common manifestations of obesity, as well as a risk factor for the development of cardiovascular disease, insulin resistance, and metabolic syndrome [49]. This is due to the fact that TGs are crucial for lipid storage and the transport of FAs in plasma [50]. Furthermore, lipid intermediates (e.g., free FAs, diacylglycerols, and ceramides) generated during TAG synthesis or lipolysis interfere with insulin signaling, which can lead to insulin resistance development [51]. Therefore, observed increases in TG concentrations in obese children require particular attention and should be an important target for obesity management to prevent the development of disease complications. 

Another class of lipids that was increased in the obese group was SMs. These are components of lipid rafts, precursors of ceramides, and other sphingolipid metabolites, and they are involved in signaling pathways [52]. Recent studies have shown that increased SM levels are associated with the development of prediabetes [53,54] and positively correlate with CE and waist-to-hip ratio in pre-diabetic men with abdominal obesity [54]. Our results are in line with studies on pediatric populations in Portugal showing elevated circulating SM concentrations in obese children [55]. This finding is especially interesting in the context of the precursor role of SMs in ceramide synthesis, known to be involved in atherosclerosis and insulin resistance development [56]. Moreover, SMs are also positively correlated with other lipids, such as CE [54]. Cholesterol esterification is a mechanism to avoid cellular toxicity due to the overabundance of unesterified cholesterol [57]. CE can be found in circulating lipoproteins in their hydrophobic cores (i.e., chylomicrons and low- and high-density lipoproteins) [57] and intracellular lipid droplets [58]. The main roles of CEs are the transport and storage of cholesterol and Fas [58]. Therefore, CE elevation in obese children observed in our work may be associated with impaired lipolysis and increased plasma FA levels and SM levels.

In our study, we also observed higher concentrations of two other lipid classes (PC and PI), consistent with a study on obese children in Portugal [55], but not with another study on Chinese adolescents [59]. PC and PI are the most abundant phospholipids in all mammalian cell membranes [60]. Lipid intermediates of PI, such as diacylglycerol or phosphatidic acid, are important lipid signaling agents, especially phosphatidylinositol-3,4,5-trisphosphate, which is required for activation of protein kinase B (Akt) involved in cell proliferation [61] and development of obesity and T2D [62]. Meanwhile, PC inhibits the activation, processing, and maturation of master regulator of de novo lipid synthesis sterol-regulatory-element-binding protein 1 (SREBP-1) [60]. Through changes in SREBP-1 target gene expression, PC modulates FAs, phospholipids, and TAG synthesis and their concentrations in plasma [60]. 

Interestingly, we found elevated concentrations of 9,10-epoxyoctadecenoic acid (9,10-EpOME), a member of the linoleic epoxide (EpOME) class, in obese children. Moreover, the concentration of 9,10-EpOME was negatively correlated with body weight and BMI. EpOMEs play roles in a variety of biological functions, including immune responses, pain perception, and cytotoxic processes [63,64,65]. 9,10-EpOME is synthesized via conversion of linoleic acid (LA), the most abundantly consumed polyunsaturated fatty acid (PUFA). The main source of LA in humans is diet, especially vegetable oils, nuts, seeds, meats, and eggs [65,66]. Through cytochrome P450-dependent metabolism, LA is converted to 9,10-EpOME, which can be metabolized by soluble epoxide hydrolase (sEH) to 9,10-dihydroxyoctadecenoic acid (9,10-DiHOME) [65]. Although knowledge on the role of EpOMEs in human metabolism remains limited, it is believed that most of the effects attributed to EpOMEs are caused by their secondary metabolites, the corresponding linoleic diols (DiHOMEs). Accumulation of DiHOMEs in the heart is associated with impaired cardiac function, including that resulting from lipopolysaccharide (LPS)-induced endotoxemic shock [67,68]. Therefore, DiHOMEs may be considered crucial metabolites mediating the toxicity of LA epoxides [69,70,71,72]. 9,10-EpOME is available from food sources such as seed oil and rice (*Oryza sativa* L.) [65], and its concentration can also be determined from the amount of linoleic acid in the diet [63].

Furthermore, elevated levels of EpOMEs can contribute to the pathology of obesity [73] and low-degree inflammation induced by obesity [64]. The limited data available for 9,10-EpOME shows that it can mediate inflammation by induction of oxidative stress in vascular endothelial cells through activation of nuclear factor kappa-light-chain-enhancer of activated B cells (NF-κB) and activator protein 1 (AP-1) transcription factors [74]. It can also lead to mitochondrial dysfunction and cell death in (rabbit) renal proximal tubules via uncoupling of oxidative phosphorylation induction [75]. Our results demonstrated decreased serum concentrations of 9,10-EpOME, which we hypothesize might be a protective mechanism during the early stage of the development of obesity to reduce immunometabolic consequences of the disease. Another possible explanation might be related to diet. LA, the main source of 9,10-EpOME, is mainly derived from food considered ‘healthy’. In children with obesity, the main cause of disease development is poor diet, and specifically high-calorie, highly-processed food lacking non-animal sources of fat, which may result in lower concentrations of LA, and hence decreased synthesis of 9,10-EpOME in obese children. However, the exact mechanism needs further investigation. 

Higher levels of BCAAs were previously observed in T2D [76,77], but few studies have focused on obesity, especially in children with obesity. Higher plasma concentrations of BCAAs were observed in Chinese adults [78], adult patients with morbid obesity [79], and older male former Finnish athletes [76], and they have been positively correlated with obesity in an Iranian adults cohort [80]. BCAA metabolites can be associated with adiposity and cardiometabolic risk during mid-childhood [81]. Furthermore, altered BCAA levels in offspring were found to be caused by maternal obesity. BCAAs were also proposed as biomarkers of metabolic syndrome [82]. Changes in BCAA concentrations can be responsible for altered plasma lipid levels through modulation of lipid metabolism. Disruption of BCAA levels can lead to impaired protein expression through changes in mammalian target of rapamycin complex1 (mTORC1) activity [83,84], and they can affect the phosphorylation level of protein kinase B (Akt), a marker this can also affect insulin signaling in muscle and liver [85,86]. Disruption of mTORC1 and Akt activity can lead to insulin resistance [83], one of the most common consequences of obesity in adults. Additionally, by activating SREBPs, both mTORC1 and Akt can modulate de novo lipogenesis [87], offering another explanation for the observed changes in circulating lipids in obese children. Therefore, our results help to reveal a novel mechanism of obesity and insulin resistance development in children associated with increased BCAA concentrations. 

Finally, we observed a significant reduction in serum phosphate concentration in children with obesity. This is in line with observations in adult obese populations, but it has not been studied extensively in pediatric patients [88,89]. Phosphorus availability modulates adenosine triphosphate (ATP) production. In patients with obesity, two main factors contribute to decreased availability of phosphorus; firstly, ‘western’ dietary patterns, including high consumption of refined cereals, potatoes, fructose, and oils, all of which negatively impact phosphorus availability; secondly, insulin resistance, one of the most common consequences of obesity, characterized by increased insulin release, which in turn stimulates the phosphorylation of many compounds and limits phosphorus availability for ATP production, exacerbating the effects of low phosphorus availability caused by diet [88]. Thus, in our study, the decreased concentration of phosphate might indicate the early stages of insulin resistance development in obese children. 

Our study has several limitations. The relatively small size of the studied groups requires relatively large effect sizes in differences in metabolites in order to infer statistical significance, especially for sex-specific ones. Moreover, in our study we used BMI as a criterion for obesity diagnosis, rather than body-fat content which would be a more accurate parameter of adiposity. However, even a body-fat criterion requires the application of an arbitrary cutoff value to classify obesity vs. non-obesity, the precise definition of which remains matter of debate in the field. Additionally, findings from untargeted metabolomic analysis would require further validation using targeted approaches. However, this study does not attempt to resolve the relative contributions of molecules to obesity development in children but instead presents whole-metabolome data on understudied pediatric populations. 

## 5. Conclusions

Herein, we present the early manifestation of obesity pathogenesis and its metabolic consequences in children using serum metabolome data, and the results reveal lipid metabolism as a key mechanism. The novel whole-metabolome approach identified different metabolites, including polar metabolites, in a pediatric population for the first time. We confirmed that findings from adult populations could be applied to pediatric populations. We identified several metabolites, including 9,10-EpOME, phosphate, and BCAAs, that contribute to obesity development, especially its consequences, which can be further investigated in mechanistic studies. Our findings will contribute to better exploration of these topics in future studies, and they will inspire future directions in the field.

## Figures and Tables

**Table 1:** Clinical characteristics of studied groups. Results are means ± standard deviation (SD). Multiple testing corrections were performed using the false discovery rate (FRD) method (*p*-value adj.). BMI, body mass index; HDL, high-density lipoprotein cholesterol; LDL, low-density lipoprotein cholesterol; TSH, thyroid-stimulating hormone; SBP, systolic blood pressure; DBP, diastolic blood pressure.

Parameter	Control Group (*n* = 15)		Obesity Group (*n* = 27)		*p*-Value	*p*-Value adj.
	Mean	SD	Mean	SD		
Age (years)	10.88	±5.14	10.41	±3.87	0.823	1
Sex (F/M)	8/7	-	15/12	-	0.910	1
BMI (kg/m^2^)	17.63	±3.19	26.91	±3.77	<0.001	<0.001
BMI Z-score	−0.45	±1.72	2.18	±0.12	<0.001	<0.001
Uric acid (mmol/L)	4.52	±0.77	4.73	±0.79	0.599	1
Creatinine (mg/dL)	0.53	±0.22	0.51	±0.12	0.665	1
Glucose (mg/dL)	88.27	±2.98	91.3	±9.49	0.792	1
Total cholesterol (mg/dL)	156.97	±10.37	166.91	±24.11	0.253	1
Triglycerides (mg/dL)	81.00	±17.13	95.81	±50.65	0.583	1
HDL (mg/dL)	51.80	±7.30	50.26	±11.95	0.407	1
LDL (mg/dL)	89.00	±13.24	95.07	±24.73	0.602	1
TSH (μIU/mL)	2.76	±0.75	2.81	±0.97	0.848	1
SBP (mmHg)	112.00	±7.42	116.37	±7.42	0.311	1
DBP (mmHg)	65.07	±6.09	68	±4.73	0.132	0.792

## Data Availability

The data that support the findings of this study are available from the corresponding author, (L.S), upon request.

## Supplementary Materials

The following supporting information can be downloaded at https://www.mdpi.com/article/10.3390/nu14010214/s1: Figure S1: Heatmap of Pearson correlation coefficient matrix between selected metabolites and clinical variables. Table S1: List of metabolites significantly differentially expressed between obesity and control groups. Table S2: ROC Area under the Curve (AUC) for each metabolite to evaluate their ability to predict patient groups.
