# Supplementary material for: Untargeted Metabolomics Analysis of the Serum Metabolic Signature of Childhood Obesity
Source: Nutrients. 2022 Jan 4;14(1):214. doi: 10.3390/nu14010214 (PMC8747180; doi:10.3390/nu14010214)
Supplement: Supplementary file 1 [file nutrients-14-00214-s001.zip › nutrients-1535926-supplementary.pdf]

Supplementary Figure S1. Heatmap of Pearson correlation coefficient matrix between selected metabolites and clinical variables. *Red and blue indicate negative and positive correlations, respectively. Color intensity indicates the absolute correlation value. Empty cells indicate non-significant correlations (adjusted p-values > 0.05). SM – sphingomyelin; PC – Phosphatidylcholine; CE - cholesteryl ester; TG 58:8 – Triacylglycerol 58:8; 9,10-EpOME - 9,10-Epoxyoctadecenoic acid; BMI – body mass index; TG – total triglycerides; HDL - high-density lipoprotein cholesterol; LDL - low-density lipoprotein cholesterol; TSH - Thyroid-Stimulating Hormone; SBP – systolic blood pressure; DBP – diastolic blood pressure.*

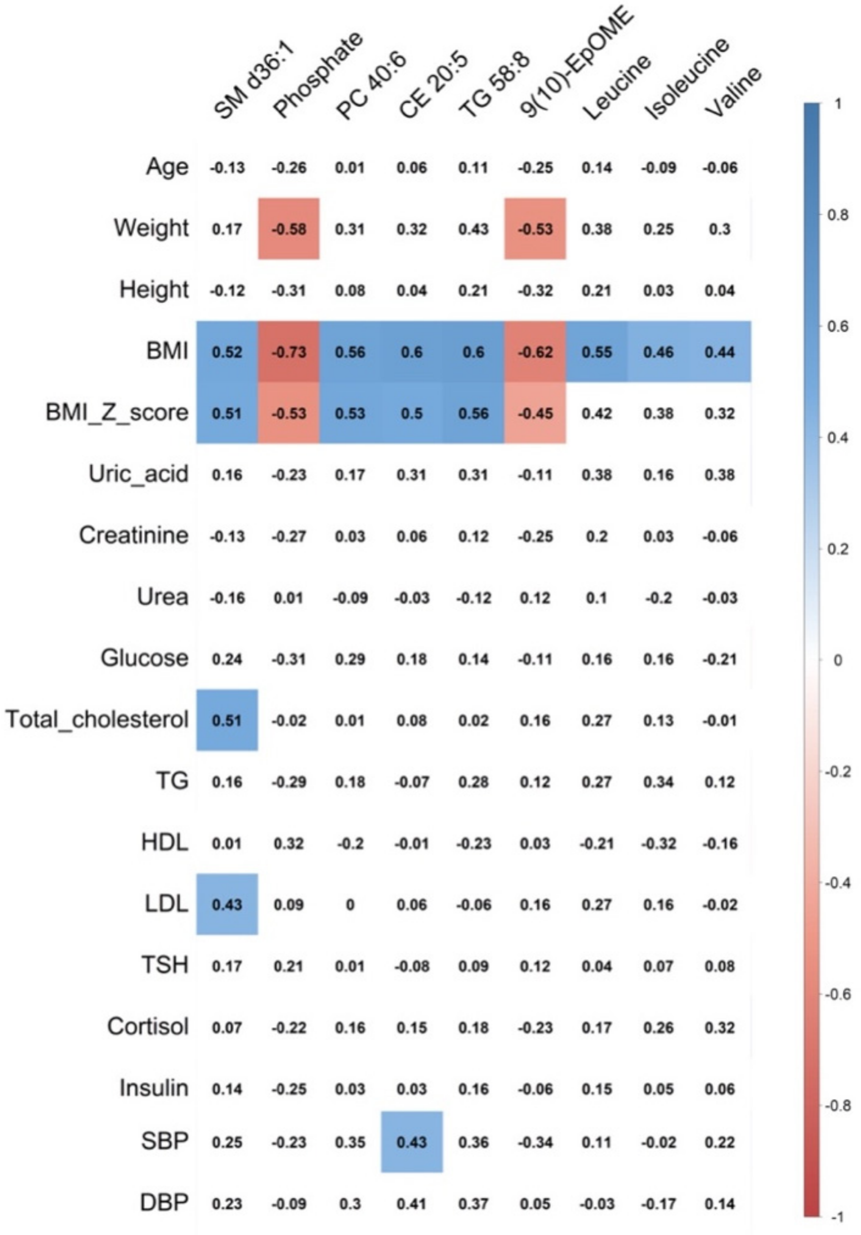

Supplementary Table S1. List of metabolites significantly differentially expressed between obesity and control groups. *Presented are metabolites for which the unadjusted p-value was <0.05, with the fold change (FC), log2 fold change (log2(FC)), p-value and adjusted p-value (adj. p-value). Multiple testing correction was done using the False Discovery Rate method. The FC was calculated by dividing the metabolite concentration in the obesity group by the concentration in the control group. SM – sphingomyelin; PC – Phosphatidylcholine; PI – Phosphatidylinositol; CE - cholesteryl ester; TG – Triacylglycerol; 9,10-EpOME - 9,10-Epoxyoctadecenoic acid; LPC – Lysophosphatidylcholine; FA – fatty acid; Cer – ceramide; G-Glu-Glu - gamma-Glutamyl-glutamic acid.*

Metabolite name	*FC*	*log2(FC)*	*p-value*	*adj. p-value*
phosphate	0.74	-0.43	6.73E-07	0.0002
SM d36:1	1.56	0.64	5.54E-07	0.0002
CE 22:6	2.05	1.03	1.09E-05	0.0015
CE 20:5	2.30	1.20	8.46E-06	0.0015
PC 40:6	2.16	1.11	3.25E-05	0.0035
9(10)-EpOME	0.54	-0.88	8.83E-05	0.0076
Phenylalanine	1.38	0.46	0.0001	0.0076
PC 36:4.1	1.38	0.46	0.0001	0.0076
Glutamine	1.46	0.54	0.0001	0.0076
PI 38:4|PI 18:0_20:4	1.51	0.59	0.0001	0.0076
PC 38:4	1.65	0.72	0.0002	0.0076
PC 36:4	1.75	0.80	0.0002	0.0076
PC P-38:4 or PC O-38:5	1.83	0.87	0.0002	0.0076
TG 58:8	2.40	1.27	0.0002	0.0076
PC 40:6.1	1.71	0.77	0.0003	0.0098
Leucine	1.23	0.30	0.0005	0.0118
beta-Alanine	1.24	0.31	0.0004	0.0118
alanine	1.43	0.52	0.0005	0.0118
PC 38:5	1.66	0.73	0.0004	0.0118
PC 40:5	1.76	0.82	0.0005	0.0118
PC 40:7	1.88	0.91	0.0005	0.0118
PC P-40:6 or PC O-40:7	1.94	0.95	0.0004	0.0118
valine	1.17	0.23	0.0006	0.0124
PC 40:5.1	1.39	0.47	0.0006	0.0124
PC 36:5	1.67	0.74	0.0006	0.0124
PC P-38:5 or PC O-38:6	1.69	0.76	0.0007	0.0148

PC P-40:4 or PC O-40:5.1	1.61	0.69	0.0008	0.0154
PC 35:4.1	1.62	0.69	0.0008	0.0154
Retinoic acid	1.28	0.36	0.0009	0.0169
PC 38:5.1	1.34	0.42	0.0010	0.0175
PE P-40:6 or PE O-40:7	1.45	0.53	0.0010	0.0175
Isoleucine	1.21	0.28	0.0012	0.0186
PC P-38:4 or PC O-38:5.1	1.45	0.53	0.0012	0.0186
PC 40:4	1.49	0.57	0.0012	0.0186
PC 37:4	1.58	0.66	0.0012	0.0186
TG 58:9	2.25	1.17	0.0012	0.0186
Methionine	1.87	0.90	0.0014	0.0202
PC 34:4	1.63	0.71	0.0015	0.0218
Lysine	1.25	0.32	0.0016	0.0223
SM d36:2	1.30	0.37	0.0017	0.0229
Androstane-3,17-diol	0.75	-0.41	0.0019	0.0238
TG 56:5	1.89	0.92	0.0019	0.0238
TG 56:7	2.13	1.09	0.0019	0.0238
PC 36:2;2O|PC 18:0_18:2;2O	0.66	-0.60	0.0021	0.0244
PE P-40:5 or PE O-40:6	1.44	0.53	0.0021	0.0244
PC 36:6	1.81	0.85	0.0021	0.0244
Serine	1.20	0.26	0.0022	0.0250
PE P-36:4 or PE O-36:5	1.73	0.79	0.0024	0.0262
PC O-36:5	1.60	0.67	0.0025	0.0263
TG 54:5	2.01	1.01	0.0025	0.0263
SM d36:0.1	1.65	0.73	0.0027	0.0271
PC 37:4.1	1.43	0.51	0.0028	0.0273
PI 36:4|PI 16:0_20:4	1.56	0.64	0.0028	0.0273
DG 36:3	1.45	0.53	0.0029	0.0274
PC P-38:5 or PC O-38:6.1	2.04	1.03	0.0029	0.0274
PE P-36:4 or PE O-36:5.1	1.63	0.70	0.0030	0.0282
threonic acid	1.31	0.39	0.0032	0.0284
SM d36:0	1.37	0.45	0.0031	0.0284
Cholesterol	0.95	-0.08	0.0035	0.0305
TG 54:4	1.62	0.69	0.0036	0.0314
SM 36:2;2O|SM 18:2;2O/18:0	1.32	0.40	0.0039	0.0332
TG 58:6	1.69	0.76	0.0039	0.0332
Ornithine	1.14	0.19	0.0044	0.0351
PE 38:6.1	1.61	0.69	0.0043	0.0351
TG 54:6	1.82	0.86	0.0043	0.0351

TG 56:8	1.88	0.91	0.0045	0.0359
Cer d36:1.1	1.26	0.34	0.0052	0.0409
FA 22:1 (erucic acid)	0.76	-0.40	0.0060	0.0444
LPC O-16:1	0.82	-0.28	0.0060	0.0444
SM d38:2.1	1.19	0.25	0.0058	0.0444
PC P-40:7 or PC O-40:8	1.44	0.53	0.0059	0.0444
PC 38:3.1	1.36	0.44	0.0065	0.0469
PC 36:5.1	1.44	0.53	0.0065	0.0469
SM d30:1.1	1.29	0.37	0.0070	0.0477
Homoarginine	1.33	0.41	0.0071	0.0477
PC 40:4.1	1.34	0.42	0.0071	0.0477
TG 53:5	1.60	0.68	0.0070	0.0477
TG 52:5	1.61	0.69	0.0069	0.0477
PC P-44:4 or PC O-44:5.1	1.35	0.43	0.0072	0.0477
PC 39:6	2.00	1.00	0.0073	0.0477
TG 53:4	1.69	0.75	0.0076	0.0495
PC P-40:4 or PC O-40:5	1.41	0.50	0.0079	0.0499
PC 38:6	1.45	0.54	0.0079	0.0499
Cer d40:2	1.26	0.33	0.0083	0.0518
His-Val	1.36	0.44	0.0090	0.0522
LPC O-16:0	0.79	-0.34	0.0090	0.0522
PC P-40:5 or PC O-40:6	1.39	0.48	0.0090	0.0522
PI 38:3 | PI 18:0_20:3	1.42	0.50	0.0090	0.0522
TG 51:3	1.51	0.59	0.0086	0.0522
TG 53:2	1.44	0.53	0.0089	0.0522
TG 53:3	1.54	0.62	0.0089	0.0522
SM d37:1	1.25	0.32	0.0093	0.0536
Hippuric acid	0.76	-0.39	0.0097	0.0547
PE P-38:5 or PE O-38:6.1	1.48	0.57	0.0097	0.0547
Cer d32:1	1.32	0.40	0.0105	0.0562
PC 37:6	1.44	0.53	0.0105	0.0562
PC P-36:3 or PC O-36:4.1	1.30	0.38	0.0105	0.0562
PC P-42:5 or PC O-42:6	1.46	0.54	0.0105	0.0562
SM d32:1.1	1.28	0.35	0.0102	0.0562
PC O-34:5	0.82	-0.29	0.0111	0.0579
PC P-36:4 or PC O-36:5	1.56	0.64	0.0111	0.0579
DG 34:2	1.32	0.40	0.0117	0.0606
G-Glu-Glu	0.83	-0.26	0.0118	0.0607
Nicotinamide	1.39	0.47	0.0122	0.0615

PC 37:3	1.21	0.28	0.0122	0.0615
aspartic acid	1.34	0.42	0.0130	0.0622
Cer d42:2.1	1.21	0.27	0.0133	0.0622
γ-Glutamylleucine	0.88	-0.19	0.0133	0.0622
PC 34:2;2O丨PC16:0_18:2;2O	0.72	-0.47	0.0130	0.0622
PC 34:4.1	1.46	0.55	0.0132	0.0622
PE 38:6	1.54	0.62	0.0131	0.0622
PE P-38:5 or PE O-38:6	1.53	0.61	0.0130	0.0622
TG 50:5	1.79	0.84	0.0132	0.0622
Phe-Phe	1.89	0.92	0.0143	0.0664
TG 52:4	1.48	0.56	0.0154	0.0710
Cer d42:2	1.20	0.26	0.0166	0.0751
PC P-44:4 or PC O-44:5	1.36	0.44	0.0166	0.0751
CE 20:4	1.30	0.38	0.0177	0.0789
Cer d39:1	1.25	0.32	0.0178	0.0789
PC P-36:3 or PC O-36:4	1.44	0.53	0.0179	0.0789
TG 50:4丨TG 14:1_18:1_18:2	1.45	0.54	0.0185	0.0810
PE 38:4.1	1.39	0.47	0.0192	0.0834
glutamyl-valine	0.82	-0.28	0.0207	0.0876
PC P-42:4 or PC O-42:5	1.32	0.40	0.0207	0.0876
PE 38:4	1.67	0.74	0.0207	0.0876
SM d36:3	1.20	0.26	0.0209	0.0880
SM d32:0	1.23	0.30	0.0213	0.0885
TG 51:2	1.40	0.48	0.0214	0.0885
Ala-Leu	1.49	0.58	0.0225	0.0927
maleimide	1.24	0.31	0.0237	0.0968
PC 38:4丨PC 18:1_20:3	1.30	0.38	0.0243	0.0977
PC P-38:3 or PC O-38:4	1.35	0.43	0.0243	0.0977
PC 16:0	0.81	-0.30	0.0255	0.1003
SM d38:1	1.17	0.22	0.0255	0.1003
TG 56:4	1.36	0.44	0.0255	0.1003
2-hydroxybutanoic acid	1.25	0.32	0.0264	0.1031
Gly-Val	1.47	0.56	0.0274	0.1039
Meprobamate	0.79	-0.35	0.0272	0.1039
PE P-40:4 or PE O-40:5	1.17	0.23	0.0274	0.1039
TG 49:2	1.52	0.61	0.0273	0.1039
TG 56:6	1.54	0.62	0.0291	0.1097
PC O-44:6	1.40	0.48	0.0314	0.1167
PE P-38:4 or PE O-38:5	1.41	0.50	0.0314	0.1167

TG 52:3	1.33	0.41	0.0324	0.1198
SM d39:2	1.21	0.28	0.0335	0.1230
asparagine	1.18	0.24	0.0358	0.1297
cysteine	1.23	0.29	0.0358	0.1297
glycolic acid	0.87	-0.20	0.0384	0.1318
LPE O-16:1	0.85	-0.23	0.0373	0.1318
Pantothenic acid	1.19	0.25	0.0383	0.1318
SM d32:2	1.31	0.39	0.0384	0.1318
TG 54:3	1.27	0.34	0.0384	0.1318
Tyrosine	1.16	0.21	0.0373	0.1318
tyrosine	1.14	0.19	0.0384	0.1318
DL-Octopamine	1.13	0.18	0.0410	0.1398
Histidine	1.11	0.15	0.0420	0.1425
CE 16:1	1.22	0.29	0.0451	0.1519
citric acid	0.85	-0.24	0.0458	0.1532
TG 49:3	1.58	0.66	0.0481	0.1600
taurine	1.58	0.66	0.0485	0.1605
PC 34:3	1.21	0.28	0.0489	0.1607
PC P-34:1 or PC O-34:2.1	0.85	-0.24	0.0497	0.1623

Supplementary Table S2. ROC Area under the Curve (AUC) for each metabolite to evaluate their ability to predict patient groups. *Univariate ROC curve analysis was done for each metabolite. Presented are the AUC values for each metabolite. SM – sphingomyelin; PC – Phosphatidylcholine; PI – Phosphatidylinositol; CE - cholesteryl ester; TG – Triacylglycerol; 9,10-EpOME - 9,10-Epoxyoctadecenoic acid; LPC – Lysophosphatidylcholine; FA – fatty acid; Cer – ceramide; G-Glu-Glu - gamma-Glutamyl-glutamic acid.*

Metabolite name	*AUC*	*Metabolite name*	*AUC*	*Metabolite name*	*AUC*
phosphate	0.972	indole-3-acetate	0.677	Urocanic acid	0.577
SM d36:1	0.928	TG 48:3	0.676	xylose	0.577
CE 20:5	0.923	histidine	0.674	PC 32:1	0.577
CE 22:6	0.918	LPC 20:1	0.673	FA 22:2 (docosadienoic acid)	0.577
PC 40:6	0.895	SM d39:1	0.672	succinic acid	0.576
9(10)-EpOME	0.872	2-aminobutyric acid	0.672	LPC 18:3	0.576
Glutamine	0.862	PC P-36:4 or PC O-36:5.1	0.672	PC P-32:0 or PC O-32:1.1	0.576
PI 38:4 | PI 18:0_20:4	0.862	PC P-36:2 or PC O-36:3	0.672	SM d42:1	0.574
PC 36:4	0.854	myo-inositol	0.669	Lenticin	0.574
Phenylalanine	0.846	ethanolamine	0.669	GlcCer d42:2.1	0.574
PC 40:6.1	0.846	SM d43:2	0.667	GlcCer d40:1.1	0.574
PC 36:4.1	0.846	LPC 20:1.1	0.667	Acetylcarnitine	0.574
PC P-38:4 or PC O-38:5	0.841	FA 20:4 (arachidonic acid)	0.667	ST 27:1;O;S	0.574
TG 58:8	0.838	PC 32:2	0.667	H-γ-glutamyl-glutamine	0.574
PC 38:4	0.838	SM d41:1	0.665	Betonicine	0.573
PC P-40:6 or PC O-40:7	0.836	SM d43:1	0.665	5'-Methylthioadenosine	0.572
PC 38:5	0.836	PE 36:4	0.664	PC O-34:0	0.569
PC 40:5	0.833	SM d30:1	0.664	3-aminoisobutyric acid	0.569
PC 40:7	0.833	TG 49:1	0.664	Stachydrine	0.569
alanine	0.832	palmitoleic acid	0.664	3-Dehydrocarnitine	0.569
Leucine	0.832	Threonine	0.664	PC 34:2.1	0.569
PC 36:5	0.828	Cotinine	0.664	PC 36:1.1	0.569
PC 40:5.1	0.828	SM 40:1;2O	0.664	mannose	0.569
valine	0.827	1H-Imidazole	0.664	PC 37:2.1	0.567
beta-Alanine	0.823	PC P-42:4 or PC O-42:5.1	0.663	2-Methylbutyryl-L-carnitine	0.567
Retinoic acid	0.815	fucose	0.663	FA 20:3	0.567
PE P-40:6 or PE O-40:7	0.813	TG 56:3	0.662	TG 46:1	0.564
PC 38:5.1	0.813	trans-4-hydroxyproline	0.662	3-Methylhistidine	0.564
PC P-38:5 or PC O-38:6	0.810	DG 34:1	0.660	FA 22:4	0.564
PC 35:4.1	0.808	TG 51:1	0.660	FA 16:1 (palmitoleic acid)	0.564
PC P-40:4 or PC O-40:5.1	0.808	PC 38:3	0.659	FA 19:1	0.564

TG 58:9	0.808	DG 36:2	0.659	PC 32:0	0.563
Isoleucine	0.808	TG 58:4	0.659	glycerol	0.563
PC 40:4	0.808	SM d34:0.1	0.659	Cer d34:1.1	0.562
PC 37:4	0.808	lyxitol	0.658	LPC 22:4	0.560
Methionine	0.804	TG 52:6	0.658	maltose	0.560
Lysine	0.800	CE 18:3	0.656	9-myristoleate	0.560
PC P-38:4 or PC O-38:5.1	0.797	PE P-34:1 or PE O-34:2.1	0.656	FA 14:0 (myristic acid)	0.560
TG 56:7	0.795	LPC 20:2.1	0.656	TG 46:2	0.559
TG 56:5	0.795	SM d39:2.1	0.656	methanolphosphate	0.559
Androstane-3,17-diol	0.795	acylcarnitine 14:2	0.656	LPC 14:0.1	0.559
PE P-40:5 or PE O-40:6	0.792	LPC 18:0.1	0.656	Cer d34:0.1	0.556
PC 36:6	0.792	Val-Arg	0.655	GlcCer d41:1	0.556
PC 34:4	0.792	TG 50:2	0.654	Cer d41:1	0.556
PC 36:2;2O|PC 18:0_18:2;2O	0.792	LPI 18:0	0.653	CE 18:2	0.556
Serine	0.791	SM 41:1;2O	0.651	citraconic acid	0.556
SM d36:2	0.790	FA 20:3 (eicosatrienoic acid)	0.651	threitol	0.554
PE P-36:4 or PE O-36:5	0.788	Creatine	0.651	FA 10:0 (capric acid)	0.554
TG 54:5	0.787	SM d36:1.1	0.651	1-Methyladenosine	0.554
PC O-36:5	0.787	(R)-Butyrylcarnitine	0.651	Proline-hydroxyproline	0.554
SM d36:0.1	0.786	SM d32:1	0.649	TG 58:2|TG 16:0_18:1_24:1	0.553
PC 37:4.1	0.785	L-Propionylcarnitine	0.649	LPC 16:0	0.553
PI 36:4|PI 16:0_20:4	0.785	PC 38:7	0.647	PC 37:2	0.551
DG 36:3	0.783	Proline	0.647	Cer d42:1	0.551
PC P-38:5 or PC O-38:6.1	0.783	TG 52:2	0.646	PE P-36:2 or PE O-36:3.1	0.549
PE P-36:4 or PE O-36:5.1	0.782	serotonin	0.646	FA 20:1 (eicosenoic acid)	0.549
threonic acid	0.781	FA 24:1 (nervonic acid)	0.646	lactamide	0.549
TG 54:4	0.777	Cer d44:1	0.645	Cer d40:1.1	0.545
TG 58:6	0.774	PC 31:0	0.645	Phe-Trp	0.544
SM d36:0	0.774	TG 48:2	0.644	3-Cyclohexyl-1,1-dimethylurea	0.544
SM 36:2;2O|SM 18:2;2O/18:0	0.774	SM d43:2.1	0.644	TG 56:2	0.544
TG 54:6	0.772	PC 38:2.1	0.644	beta-alanine	0.542
Cholesterol	0.772	Hexaethylene glycol	0.644	Bilirubin	0.541
PE 38:6.1	0.771	acylcarnitine 10:1	0.644	PC 28:0	0.541
TG 56:8	0.769	TG 47:1|TG 15:0_16:0_16:1	0.641	LPC 22:6	0.541
Ornithine	0.768	SM d40:3	0.641	oleic acid	0.540
Cer d36:1.1	0.765	LPC 20:2	0.641	FA 20:2 (eicosadienoic acid)	0.538
SM d38:2.1	0.763	SM d38:0	0.641	PC 35:2.1	0.538
FA 22:1 (erucic acid)	0.762	SM d42:3.1	0.640	Caffeine	0.537
PC P-40:7 or PC O-40:8	0.762	Tryptophan	0.640	GlcCer d42:1	0.536

LPC O-16:1	0.762	Betaine	0.640	TG 51:0 I TG 16:0_17:0_18:0	0.536
PC 36:5.1	0.759	PC P-40:3 or PC O-40:4	0.638	PC 35:1	0.536
PC 38:3.1	0.759	LPC 18:1	0.638	Citrulline	0.536
SM d30:1.1	0.756	SM d33:1	0.638	TG 58:3	0.536
PC 40:4.1	0.756	PC O-34:1	0.638	PC 33:0	0.535
Homoarginine	0.756	4-Guanidinobutyric acid	0.638	PC 35:1.1	0.535
TG 52:5	0.756	LPC 18:0	0.638	LPE 22:6	0.535
TG 53:5	0.756	SM d38:1.1	0.637	Cer d34:2	0.533
PC 39:6	0.755	Nudifloramide	0.636	3-Amino-1-propanol	0.533
TG 53:4	0.754	LPC 18:2.1	0.636	LPC 20:3.1	0.533
PC P-44:4 or PC O-44:5.1	0.751	FA 22:5	0.636	LPE 18:0.1	0.533
Cer d40:2	0.751	Arg-Phe	0.635	PC 36:3	0.533
TG 51:3	0.750	PC 33:1	0.635	Didodecyl 3,3'-thiodipropionate	0.533
PC P-40:4 or PC O-40:5	0.749	isocitric acid	0.635	lactic acid	0.533
TG 53:3	0.749	Arginine	0.633	PC O-42:9	0.532
PC P-40:5 or PC O-40:6	0.749	PC 34:3.1	0.633	TG 50:0	0.532
TG 53:2	0.749	SM d41:2	0.633	PE 36:2 I PE 18:0_18:2	0.532
His-Val	0.749	Cer d43:1	0.632	PI 34:1 I PI 16:0_18:1	0.531
LPC O-16:0	0.749	arabitol	0.632	LPE 20:4	0.531
PC 38:6	0.749	TG 48:4	0.631	PC 34:2	0.531
PI 38:3 I PI 18:0_20:3	0.749	PC 33:2.1	0.629	LPC 22:5	0.531
PE P-38:5 or PE O-38:6.1	0.746	PC 33:2	0.629	TG 48:0	0.528
Hippuric acid	0.746	SM d34:2	0.628	PC 36:4;2O I PC 16:0_20:4;2O	0.528
PC 37:6	0.744	SM d40:1	0.628	Cer 18:2;2O/22:0	0.528
PC P-42:5 or PC O-42:6	0.744	Cer d33:1	0.628	erythritol	0.528
SM d37:1	0.744	SM d34:0	0.626	HMBA	0.528
PC P-36:3 or PC O-36:4.1	0.744	SM d33:1.1	0.626	FA 18:1 (oleic acid)	0.528
Cer d32:1	0.744	acylcarnitine 10:0	0.626	Urea	0.528
SM d32:1.1	0.741	PC 32:2.1	0.626	LPC 20:3	0.527
DG 34:2	0.740	LPC 20:5	0.624	GlcCer d42:2	0.526
L-γ-Glutamyl-L-glutamic acid	0.740	LPC 16:1	0.622	SM d34:1.1	0.526
PC P-36:4 or PC O-36:5	0.738	PI 36:1 I PI 18:0_18:1	0.621	PE P-36:2 or PE O-36:3	0.526
PC 37:3	0.738	acylcarnitine 18:1	0.621	N-Methylproline	0.526
Nicotinamide	0.738	PC P-40:3 or PC O-40:4.1	0.618	PC 34:0.1	0.523
PC O-34:5	0.738	LPC 18:2	0.618	PC 36:2.1	0.523
PE 38:6	0.736	oxoproline	0.618	PC O-32:0	0.523
PC 34:4.1	0.736	5-Aminovaleric acid	0.617	FA 17:0	0.523
TG 50:5	0.736	Cer d40:0	0.615	Cer d42:1.1	0.523
γ-Glutamylleucine	0.736	allantoic acid	0.615	FA 15:0 (pentadecylic acid)	0.522

Cer d42:2.1	0.736	isothreonic acid	0.615	PE 34:2.1	0.522
Phe-Phe	0.733	LPC 14:0	0.615	CE 18:1	0.521
aspartic acid	0.733	PC P-42:3 or PC O-42:4	0.614	glyceric acid	0.521
PE P-38:5 or PE O-38:6	0.733	5-methoxytryptamine	0.613	PC 32:0.1	0.521
PC 34:2;2O∣PC 16:0_18:2;2O	0.733	FA 17:1	0.613	Octanoylcarnitine	0.521
4-Aminobenzoic acid	0.732	Erucamide	0.610	acylcarnitine 16:0	0.521
TG 52:4	0.731	Isovaleryl-L-carnitine	0.610	TG 54:1	0.521
Cer d42:2	0.728	3-Indolepropionic acid	0.608	ribose	0.521
PC P-36:3 or PC O-36:4	0.726	TG 54:2	0.608	3-Hydroxybutyrylcarnitine	0.519
PC P-44:4 or PC O-44:5	0.726	SM d44:2	0.608	TG 52:1	0.518
Cer d39:1	0.726	FA 14:1 (physeteric acid)	0.608	Hypoxanthine	0.518
CE 20:4	0.724	LPC 16:1.1	0.608	Carnitine	0.518
TG 50:4∣TG 14:1_18:1_18:2	0.724	PC 36:1	0.608	GlcCer d40:1	0.518
PE 38:4.1	0.723	PC 30:0	0.608	LPC 15:0	0.518
PC P-42:4 or PC O-42:5	0.721	PC P-32:1 or PC O-32:2	0.606	biuret	0.518
PE 38:4	0.721	Octadecanedioic acid	0.605	TG 56:1∣TG 18:0_20:0_18:1	0.518
glutamyl-valine	0.721	LPE 18:2	0.605	PC 34:1.1	0.518
SM d32:0	0.719	acylcarnitine 18:2	0.605	Cer 18:1;2O/24:0	0.518
TG 51:2	0.719	indole-3-propionic acid	0.603	acylcarnitine 18:0	0.517
SM d36:3	0.718	LPE 18:0	0.603	PC O-32:0.1	0.517
Ala-Leu	0.715	Choline	0.603	PC P-34:0 or PC O-34:1	0.515
maleimide	0.715	Cer d40:1	0.603	SM d34:1	0.515
TG 56:4	0.713	Creatinine	0.603	PC 32:1.1	0.515
SM d38:1	0.713	LPC 18:1.1	0.601	SM d42:2	0.515
PC 38:4∣PC 18:1_20:3	0.713	His-Trp	0.600	Kynurenine	0.515
PC P-38:3 or PC O-38:4	0.713	SM d34:2.1	0.600	PE P-34:1 or PE O-34:2	0.515
PC 16:0	0.713	acylcarnitine 12:0	0.600	N-Methylhistidine	0.515
2-hydroxybutanoic acid	0.712	PI 34:2∣PI 16:0_18:2	0.600	TMAO	0.515
TG 49:2	0.710	pyruvic acid	0.599	PC P-34:2 or PC O-34:3	0.514
Gly-Val	0.710	TG 53:1	0.597	aminomalonate	0.513
Meprobamate	0.710	Imidazole	0.597	Decanoyl-L-carnitine	0.513
PE P-40:4 or PE O-40:5	0.710	sorbitol	0.596	PC 36:3.1	0.513
TG 56:6	0.708	cystine	0.595	PI 36:2∣PI 18:0_18:2	0.513
PC O-44:6	0.705	4-Acetamidobutyric acid	0.595	LPE 16:0	0.513
PE P-38:4 or PE O-38:5	0.705	FA 18:3 (linolenic acid)	0.595	alpha-Methylhistidine	0.513
TG 52:3	0.703	TG 58:1∣TG 16:0_24:0_18:1	0.594	LPC 17:1	0.513
SM d39:2	0.703	Cer d38:1.1	0.592	LPC 20:4	0.512
cysteine	0.700	GlcCer d38:1	0.592	Cer d34:0	0.510
asparagine	0.700	TG 48:1	0.590	PC P-34:1 or PC O-34:2	0.510

SM d32:2	0.697	fructose	0.590	indole-3-lactate	0.510
Pantothenic acid	0.697	glycine	0.590	FA 18:2 (linoleic acid)	0.510
Tyrosine	0.697	PC P-40:1 or PC O-40:2	0.590	GlcCer d42:1.1	0.508
glycolic acid	0.697	PC 32:3	0.590	PC 36:2	0.508
TG 54:3	0.697	CE 20:3	0.590	Cer d34:1	0.508
LPE O-16:1	0.697	Phenylacetyl-L-glutamine	0.588	PC P-36:2 or PC O-36:3.1	0.506
DL-Octopamine	0.695	3-hydroxybutyric acid	0.588	Cer d42:0	0.505
Histidine	0.694	Leu-Leu	0.587	TG 44:1	0.505
CE 16:1	0.691	linoleic acid	0.587	pseudo uridine	0.505
citric acid	0.690	SM d40:0	0.587	PC 33:1.1	0.504
TG 49:3	0.688	LPE 18:2.1	0.587	L- beta -Homoserine	0.504
PC P-34:1 or PC O-34:2.1	0.687	DG 30:5	0.587	uric acid	0.503
taurine	0.687	3-Aminotyrosine	0.586	PC P-38:2 or PC O-38:3	0.503
PC 34:3	0.687	PC 35:2	0.585	PE 34:1	0.503
His-Ala	0.685	TG 50:1	0.582	TG 44:0	0.503
FA 20:5	0.685	TG 49:0	0.582	Glycerophosphocholine	0.503
PC P-34:2 or PC O-34:3.1	0.685	Theobromine	0.582	Methacholine	0.503
TG 50:3	0.682	TG 46:0	0.581	PC P-32:0 or PC O-32:1	0.503
PC P-36:1 or PC O-36:2	0.682	PC 38:2	0.579	Glutamic acid	0.503
LPC 20:0	0.682	TG 47:0 | TG 14:0_16:0_17:0	0.578	PC 34:1	0.503
PC O-40:10	0.682	SM d42:1.1	0.578	Pyroglutamic acid	0.503
Asp-Phe	0.681	FA 22:6	0.578	PC 18:0_20:4;3O	0.501
p-cresol	0.681	Cer d41:1.1	0.578	Cyclo(Leu-Pro)	0.501
SM d38:2	0.679	PC 35:3	0.577	PE P-36:1 or PE O-36:2	0.500
SM d42:3	0.679	LPC 20:4.1	0.577	PC 34:0	0.500
FA 12:0 (lauric acid)	0.677	glucose	0.577		
